# Area Scaling of Nanosecond Switching Delay in HZO Capacitors with an Ultrathin ZrO_2_ Interlayer

**DOI:** 10.3390/mi17101140

**Published:** 2026-09-30

**Authors:** Liwei Hao, Yunlei Zhong

**Affiliations:** 1 School of Petrochemical Engineering, Changzhou University, No. 21, Gehu Middle Road, Wujin District, Changzhou 213164, China; 2 Key Laboratory of Multifunctional Nanomaterials and Smart Systems, Division of Advanced Materials, Suzhou Institute of Nano-Tech and Nano-Bionics, Chinese Academy of Sciences, Suzhou 215123, China

**Keywords:** hafnium zirconium oxide, ferroelectric capacitor, zirconia interlayer, switching delay, area scaling, pulse characterization, interface engineering

## Abstract

Interfacial engineering can modify the electrical response of hafnium zirconium oxide capacitors, but separating polarization dynamics from circuit charging remains essential. We compare approximately 10 nm thick Hf_0.5_Zr_0.5_O_2_ (HZO) capacitors with otherwise identical capacitors containing an additional approximately 1 nm thick ZrO_2_ bottom interlayer (hereafter HZO–ZrO_2_ capacitors). Polarization–voltage measurements, cross-sectional scanning transmission electron microscopy, capacitance–voltage characterization, and switching/non-switching pulse measurements were combined across electrode areas of 25–10,000 μm^2^. Both stacks exhibit ferroelectric hysteresis and butterfly-shaped capacitance curves. At 2.5 V and 100 kHz, the HZO–ZrO_2_ capacitors have 10–15% higher capacitance. Their measured switching-onset delays are nevertheless lower at areas ≥ 400 μm^2^: 2.1, 5.6, and 23.0 ns at 400, 2500, and 10,000 μm^2^, compared with 3.2, 7.7, and 30.9 ns for HZO; the largest relative reduction is 34.4%, observed at 400 μm^2^, where the delay decreases from 3.2 ns to 2.1 ns. At 25 and 100 μm^2^, the measured differences (0.1 ns) are within the ≈0.1 ns timing resolution of the single-record measurement and are reported as descriptive values. Dividing the measured delay by the capacitance yields a quantity with resistance units that serves as a useful diagnostic for comparing devices, but it cannot be equated with a physical contact resistance. In particular, simply setting the delay equal to *R* × *C* with the nominal 100 Ω external resistance leads to an inconsistency for the largest HZO–ZrO_2_ capacitors, where it would imply a negative additional resistance. These results associate ZrO_2_ insertion with reduced operational delay while establishing the calibration requirements for interpreting its physical origin.

## 1. Introduction

The discovery of ferroelectricity in hafnium oxide opened a route to polarization-based devices using materials already familiar to semiconductor manufacturing [1]. Ferroelectric behavior in the HfO_2_–ZrO_2_ system subsequently established composition as an additional control parameter [2]. Hafnium zirconium oxide, particularly Hf_0.5_Zr_0.5_O_2_ (HZO), is therefore investigated for embedded memories and computing devices that require thin active layers and electrical programmability [3]. However, a useful material response must ultimately be delivered through electrodes, contacts, and interconnects. The time required to apply a switching field can be as consequential as the polarization reversal that follows.

The polar phase of hafnia is metastable, making the response sensitive to processing and boundary conditions. Calculations identify competing bulk and surface contributions to phase stability [4], while experiments show that film thickness and annealing affect the phase mixture and dielectric response [5]. Ferroelectricity in ultrathin films directly integrated with silicon illustrates the considerable potential of thickness scaling [6]. Lateral scaling poses a separate question: reducing capacitor area lowers the charge demand, but can also change the relative contributions of the contacts, electrode spreading resistance, and measurement parasitics. Thickness and area effects should consequently be distinguished when discussing faster operation.

Interfaces participate in both structural stabilization and electrical boundary conditions. Photoelectron measurements of TiN/HZO stacks identify electrode oxidation and oxygen scavenging near the interface [7]. Operando microscopy further connects oxygen migration with structural changes in hafnia devices [8], and atomic-resolution work demonstrates reversible transformations between polar and antipolar arrangements during electrical cycling [9]. These findings make interface engineering physically well motivated. They also caution against assigning every electrical improvement to one mechanism: phase populations, local fields, screening, and defect distributions can change together.

ZrO_2_ is a particularly relevant interlayer because of its chemical and structural relationship to HZO. Seed-layer experiments report improved ferroelectricity and a role in film nucleation [10]. Related work on silicon-supported stacks associates ZrO_2_ insertion with preferential formation of the polar structure [11], while pre-annealing studies show that the interlayer thermal history matters [12]. Interface termination in epitaxial HZO likewise changes ferroelectric phase stabilization [13]. The strong influence of the bottom electrode in dimensionally scaled capacitors reinforces the need to examine the entire stack [14]. Layered HfO_2_–ZrO_2_ structures have also been investigated specifically for low-voltage, fast switching [15].

A second requirement is a consistent definition of switching time. Pulse measurements on HZO can be described using distributions of local switching times [16]. Direct transient measurements identify parasitic charging as an important limit on the apparent response [17], and optimized crossbar structures have enabled sub-nanosecond switching studies [18]. Area-dependent measurements of TiN/HZO/TiN capacitors similarly show that interfacial electrical behavior affects the measured coercive field and switching response [19]. Estimating resistance from ferroelectric hysteresis is itself an established approach [20]; a new application must therefore demonstrate its assumptions and additional information rather than claim a universally new resistance measurement.

Here, we compare HZO and HZO–ZrO_2_ capacitors over five electrode areas using the same measurement framework. The objective is to establish how the interlayer affects the observed onset delay and how that change relates to capacitance. We combine structural images, frequency-dependent hysteresis, small-signal capacitance, and paired pulse transients. Beyond comparing the two stacks, we explicitly test the consistency of a simple *RC* interpretation. This reveals both a practical delay reduction and an important distinction between an effective transient metric and a separately identifiable physical contact resistance. Our focus on the onset delay is motivated by ultrashort-pulse write operation: charging to the switching threshold consumes part of the pulse budget before any reversal can occur, making this interval a primary target for stack and interface engineering.

## 2. Materials and Methods

### 2.1. Capacitor Structures and Structural Characterization

Two metal–ferroelectric–metal capacitor structures were investigated on oxidized silicon substrates with tungsten electrodes. The reference structure contained an approximately 10 nm-thick HZO layer. The modified structure included an approximately 1 nm-thick ZrO_2_ layer between the bottom tungsten electrode and the approximately 10 nm-thick HZO film. The bottom tungsten electrodes (30 nm) were deposited on the oxidized silicon substrates by sputtering and patterned by optical lithography. The approximately 10 nm HZO film—and, for the modified structure, the approximately 1 nm ZrO_2_ interlayer—was grown by thermal atomic layer deposition (ALD) at 280 °C, using tetrakis(ethylmethylamido)hafnium and tetrakis(ethylmethylamido)zirconium as the Hf and Zr precursors and ozone as the oxygen source, with alternating HfO_2_ and ZrO_2_ cycles in a 1:1 ratio and a growth rate of approximately 1 Å per cycle. The stack was crystallized by rapid thermal annealing at 400 °C for 60 s under a sputtered tungsten capping layer. The top tungsten electrodes were then deposited by sputtering and defined by optical lithography and wet etching. A related tungsten-electrode fabrication platform is described by Hu et al. [21]. The electrode overlap defined active areas of 25, 100, 400, 2500, and 10,000 μm^2^. These areas correspond to equivalent square side lengths of 5, 10, 20, 50, and 100 μm; the films are nanoscale in thickness, whereas the measured lateral dimensions are microscale.

A related tungsten-electrode fabrication platform is described by Hu et al. [21]. Cross-sectional high-angle annular dark-field scanning transmission electron microscopy (HAADF-STEM) was used to inspect layer continuity and approximate thickness. Atomic-resolution integrated differential phase-contrast STEM (iDPC-STEM) images were examined within the selected regions to assess local lattice order. Structural interpretation is restricted to the sampled cross-sections. No phase-fraction refinement, oxygen-vacancy concentration, or statistical texture distribution is inferred from these images.

### 2.2. Electrical Measurements and Operational Definitions

Fast-pulse measurements used a pulse generator, the capacitor under test, and a 12-bit high-definition oscilloscope (WavePro HD, Teledyne LeCroy, Chestnut Ridge, NY, USA) in a series sensing configuration. The nominal generator output resistance and oscilloscope input termination were each 50 Ω, giving an external resistance *R*_ext_ of 100 Ω. The general pulse-based reconstruction strategy follows previous high-frequency ferroelectric measurements [22]. For a measured voltage *V*_s_ across the oscilloscope termination, the terminal current is *I*(t) = *V*_s_(t)/50 Ω. Reconstructing capacitor voltage additionally requires the applied source waveform and the voltage drops along the current path. A programmed generator amplitude alone does not establish the instantaneous field across the ferroelectric layer.

Polarization–voltage loops were examined from 1 kHz to 1 MHz. Capacitance–voltage curves were measured with a 100 kHz small-signal excitation, and capacitance at a positive bias of 2.5 V was selected as a common comparison point. This quantity is denoted *C*_2.5_ throughout. It represents the measured differential capacitance of the complete stack at the specified bias and frequency; it is not assumed to be an intrinsic, frequency-independent HZO capacitance. Fast dielectric characterization outside ferroelectric hafnia also illustrates the value of comparing capacitance, losses, and transient behavior under explicitly defined conditions [23].

**Figure 1:**
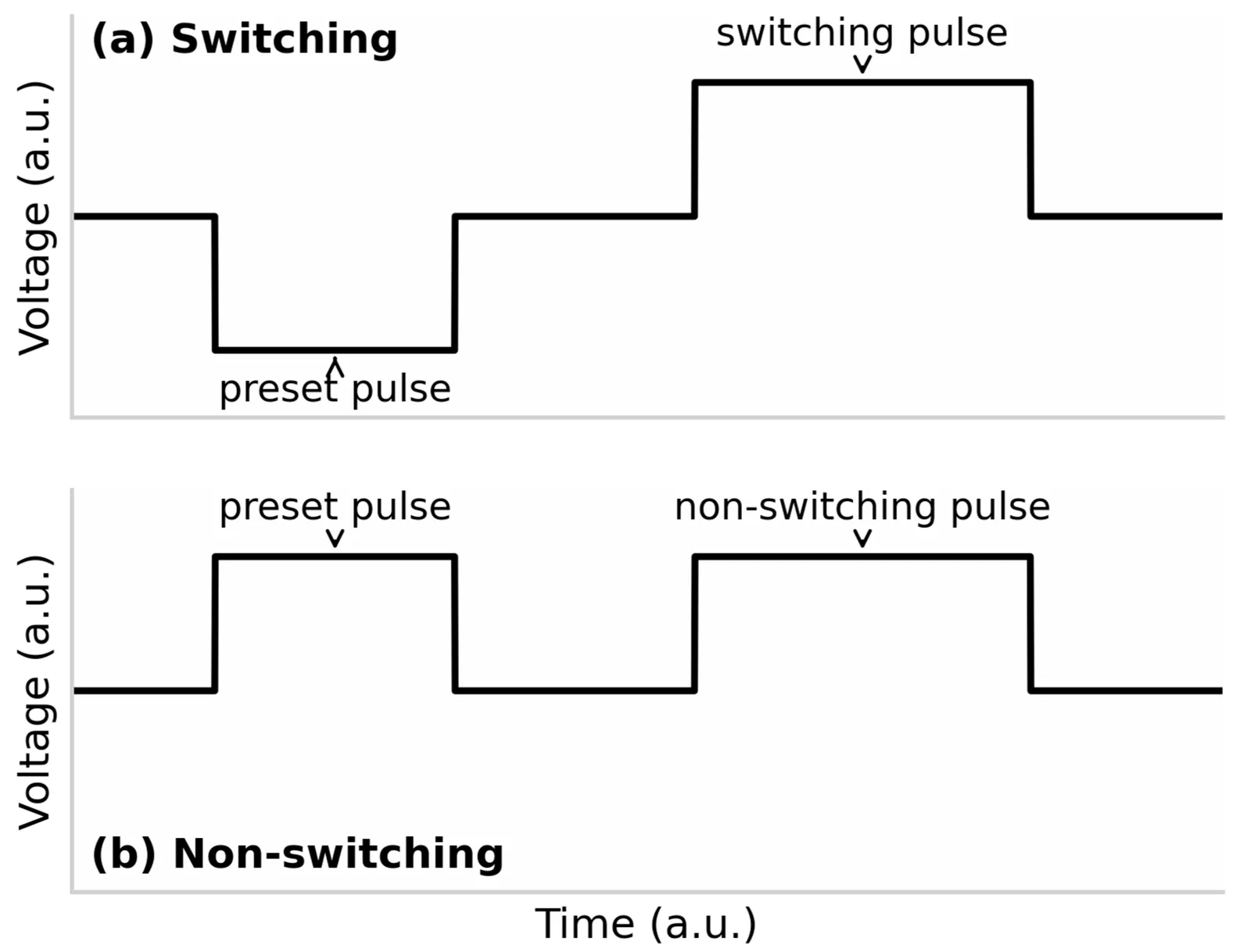
Schematic of the applied pulse sequences. (**a**) Switching measurement: a negative preset pulse initializes the polarization to the opposite state, followed by a positive switching pulse. (**b**) Non-switching measurement: a positive preset pulse initializes the polarization to the matching state, followed by an identical positive non-switching pulse. The differential polarization is obtained as Δ*P*(t) = A^−1^∫[*I*_sw_(t) − *I*_non-sw_(t)]dt, where *I*_sw_ and *I*_non-sw_ are the current responses to the switching and non-switching pulses, respectively. The pulse onset *t*_0_ is defined at the rising edge common to both pulses, and *t*_onset_ is defined at the onset of the rise of Δ*P*(t); the switching-onset delay is *t*_sd_ = *t*_onset_ − *t*_0_.

The measurement uses the two pulse sequences shown in Figure 1. For the switching measurement, a negative preset pulse initializes the polarization to the opposite state, followed by a positive switching pulse; for the non-switching measurement, a positive preset pulse initializes the polarization to the matching state, followed by an identical positive pulse. The differential signal isolates the polarization-associated contribution to the extent that the two measurements share the same charging and leakage background: subtracting the non-switching current *I*_non-sw_ from the switching current *I*_sw_ removes the charging contribution common to both pulses, and the operational switched-polarization increment is Δ*P*(t) = *A*^−1^∫[*I*_sw_(t) − *I*_non-sw_(t)]dt, with being *A* the electrode area and the integration referenced to the pulse event. Its saturated value is conventionally designated as 2*P*_r_ for a complete reversal between equivalent remanent states. Differences in nonlinear capacitance or state-dependent leakage can leave residual contributions after subtraction.

The switching-onset delay is defined as *t*_sd_ = *t*_onset_ − *t*_0_, where *t*_0_ is the time point of the rising edge of the switching and non-switching pulses, common to both measurements, and *t*_onset_ is the time point at which the Δ*P*(t) curve begins to rise, corresponding to the onset of ferroelectric domain reversal. It is distinct from the subsequent time needed to complete reversal. Because the onset is read from the separation of a paired switching/non-switching record rather than from an absolute amplitude level, the criterion is differential and is insensitive to the charging background common to both traces. *t*_sd_ is an interval within each record and excludes the subsequent polarization rise, consistent with related HZO–ZrO_2_ pulse measurements [24]. The transients were recorded with a 12-bit high-definition oscilloscope (WavePro HD, Teledyne LeCroy). The onsets were marked graphically under an identical procedure for all ten records (five areas, two stacks) and cross-checked by both authors; the markings agreed within one marking step (0.1 ns), which is also the numerical resolution of the values in Table 1. The sampling interval is 50 ps at 20 GS/s (100 ps in four-channel operation at 10 GS/s), the bandwidth-limited rise time is no faster than ≈44 ps, and the baseline noise is approximately 0.7 mV RMS at 50 mV/div sensitivity; combining these contributions gives a conservative single-reading timing uncertainty *σ*_t_ ≈ 0.1 ns. One transient record is available per area and stack; consequently, no error bars or significance tests are assigned, and differences at or below *σ*_t_ are reported as descriptive values only.

### 2.3. Threshold-Charging Model and Consistency Analysis

Before substantial switching, an idealized capacitor *C* driven by a voltage step *V*_step_ through a total positive series resistance *R*_tot_ charges according to Equation (1). If switching becomes detectable when its voltage reaches a threshold *V*th, the associated delay is given by Equation (2). Physically, *V*th corresponds to the coercive voltage of the stack under the applied pulse condition, so that *t*_sd_ is, to first order, the *RC* charging time required to raise the ferroelectric voltage from zero to the switching threshold. During this interval no domain reversal occurs, yet it consumes part of the applied pulse width; the intrinsic polarization-switching time refers to the subsequent reversal process and is not included in *t*_sd_. Measuring *t*_sd_ therefore quantifies the fraction of an applied pulse that is consumed by charging before polarization reversal can begin. Equation (1) states that, after the voltage step is applied, the capacitor voltage rises exponentially from zero toward *V*_step_ with the time constant *R*_tot_*C*. Equation (2) is obtained from Equation (1) by solving for time at which *V*_C_ first reaches the switching threshold *V*th. The dimensionless factor *α* = −ln(1 − *V*th/*V*_step_) compresses the threshold condition into a single number: for example, *V*th = 0.632 *V*_step_ gives *α* = 1, so that *t*_sd_ equals exactly one *RC* time constant, whereas a lower threshold gives *α* < 1 and a proportionally shorter delay. These relations are used here to convert the measured delay into the diagnostic metric *R*_app_ = *t*_sd_/*C* and to test how far a simple *RC* description remains consistent (Section 3.4). Equations (1)–(3) are circuit relations used here for interpretation, not fitted evidence for a unique microscopic switching mechanism.

*V*_C_ (t) = *V*_step_ [1 − exp(−t/(*R*_tot_ *C*))] (1)

*t*_sd_ = *αR*_tot_ *C*; *α* = −ln(1 − *V*th/*V*_step_) (2)

*R*_tot_ = *R*_ext_ + *R*_add_; *R*_add_ = *t*_sd_/(*αC*) − *R*_ext_ (3)

For transparent comparison, we define *R*_app_ = *t*_sd_/*C*_2.5_ and the unity-factor residual *R*_res_ = *R*_app_ − 100 Ω. Both have resistance units, but neither is automatically a contact resistance. A finite source rise time, nonlinear capacitance, distributed impedance, and the operational onset criterion can alter the relation between *R*_app_ and *R*_tot_. In particular, a negative *R*_res_ indicates failure of the unity-factor interpretation; it does not establish a negative physical resistor. We retain these signed diagnostic values rather than force them to zero or substitute undocumented positive values.

## 3. Results

### 3.1. Frequency-Dependent Hysteresis and Local Structure

**Figure 2:**
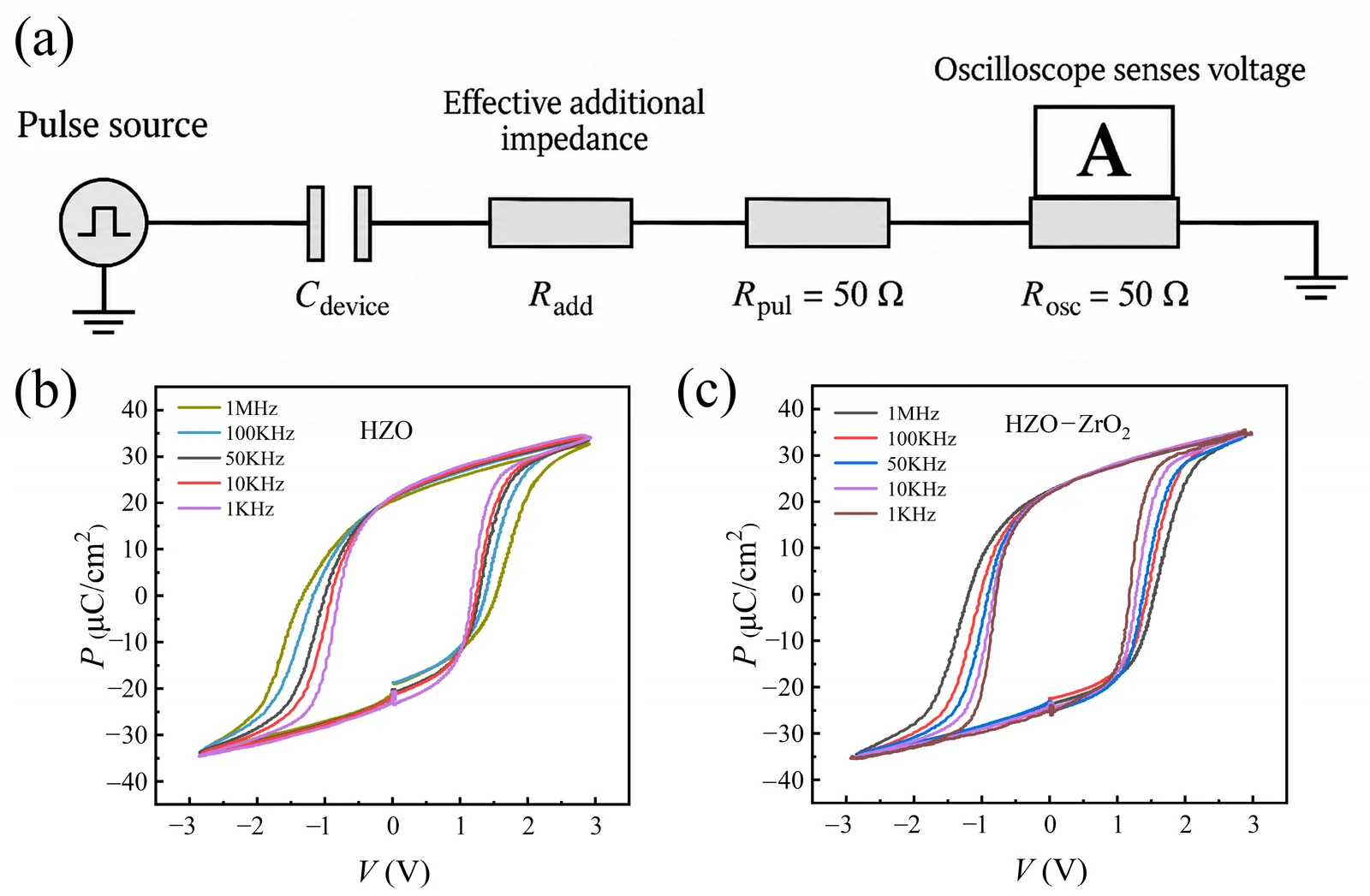
Fast-pulse characterization of hafnium zirconium oxide (HZO) capacitors. (**a**) Lumped measurement model with device capacitance, an effective additional series contribution *R*_add_, and nominal 50 Ω generator and oscilloscope resistances. *R*_add_ is not localized to a particular interface by this model. (**b**,**c**) Polarization–voltage loops for HZO and HZO–ZrO_2_, respectively, at frequencies from 1 kHz to 1 MHz.

Figure 2 shows the measurement arrangement and polarization–voltage loops. Both stacks exhibit hysteretic polarization responses across the investigated frequency range, supporting ferroelectric switching. The HZO–ZrO_2_ loops show comparatively sharper reversal regions. This is consistent with a more concentrated voltage interval for the measured reversal, although it does not independently quantify switching efficiency, energy consumption, or the distribution of microscopic nucleation barriers. Such distinctions matter because a loop contains both polarization evolution and the response of the surrounding circuit.

For both structures, the apparent coercive voltage increases as the measurement frequency increases. A higher drive is therefore required to achieve comparable reversal on a shorter timescale. Field-dependent switching is well established in ferroelectrics, beginning with classical domain-motion measurements [25]. In polycrystalline films, distributed nucleation probabilities and local fields provide additional routes to time-dependent switching [26,27]. The observed frequency dependence is compatible with these processes and with series-voltage losses. Without independently reconstructed device voltage, it cannot uniquely separate intrinsic field acceleration from extrinsic charging.

**Figure 3:**
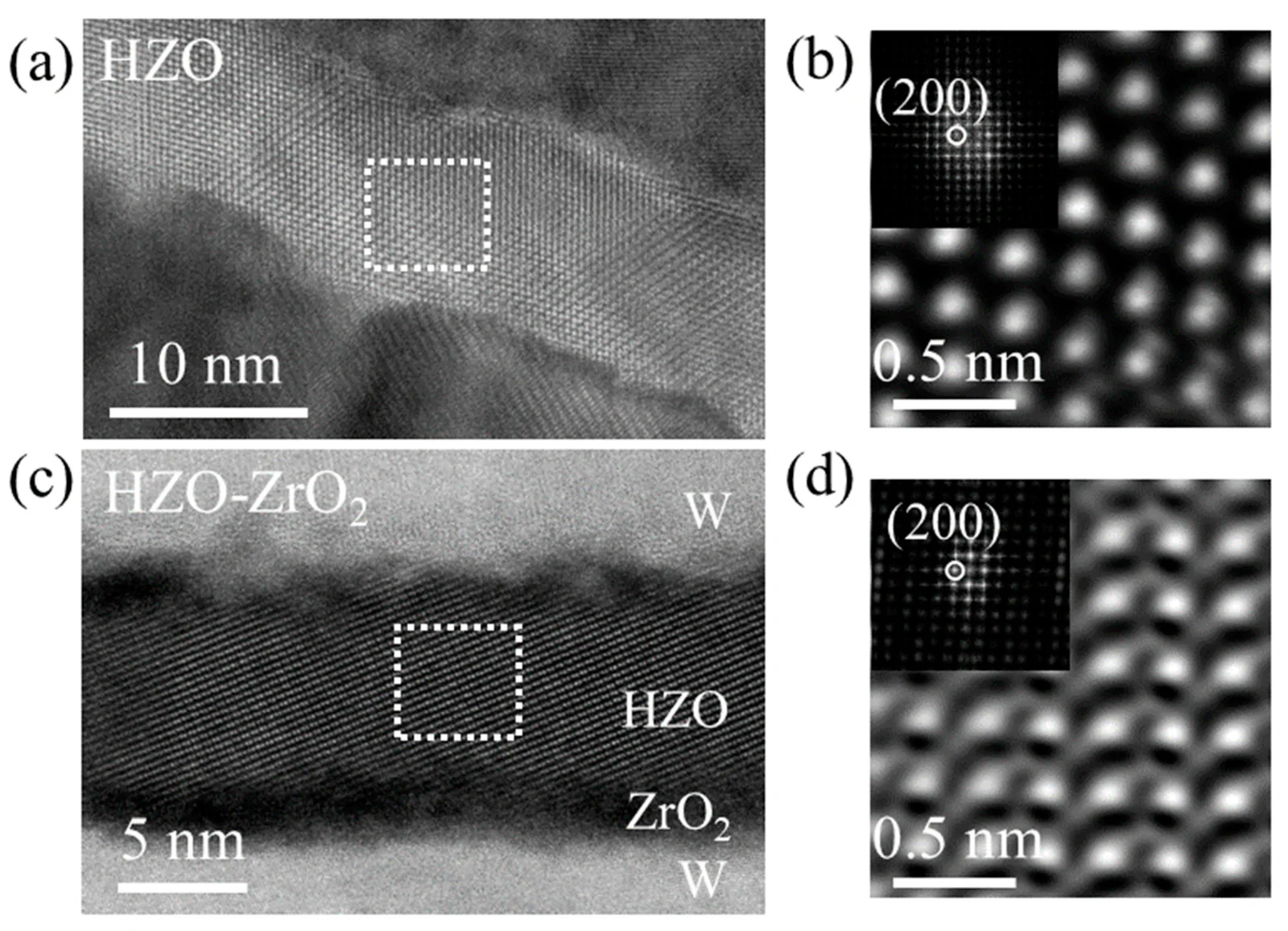
Cross-sectional scanning transmission electron microscopy of the two stacks. (**a**) HAADF-STEM image of HZO. (**b**) Atomic-resolution iDPC-STEM image of the selected region in (**a**). The inset shows the corresponding fast Fourier transform (FFT) pattern, with the diffraction spot indexed to the (200) lattice planes of crystalline HZO. (**c**) HAADF-STEM image of HZO–ZrO_2_ with the bottom ZrO_2_ region identified. (**d**) Atomic-resolution iDPC-STEM image of the selected region in (**c**). The inset shows the corresponding FFT pattern with the (200) diffraction spot marked. Scale bars are retained in each panel.

The cross-sectional images in Figure 3 show a crystalline HZO layer of approximately 10 nm in the reference capacitor. Ordered lattice contrast is resolved in the enlarged region. The modified stack contains a comparably thick HZO layer and a thin region identified as the inserted ZrO_2_ layer near the bottom tungsten electrode. Its atomic-resolution image likewise resolves local crystalline order. The fast Fourier transform (FFT) patterns of these atomic-resolution images, shown as insets in the upper-left corners of Figure 3b,d, exhibit sharp, discrete diffraction spots rather than diffuse halos, confirming well-defined lattice periodicity of the HZO layers in both stacks. The spot marked in each inset is indexed to the (200) lattice planes of crystalline HZO, which further supports the crystalline phase assignment. These observations support successful fabrication of the intended layered structures. They do not alone establish a film-wide *c*-axis orientation, a quantitative orthorhombic phase fraction, or the chemical abruptness of every interface. The electrical comparison is therefore interpreted alongside local structural evidence without equating visible crystallinity with complete phase identification.

### 3.2. Capacitance Increases with Area and with Interlayer Insertion

**Figure 4:**
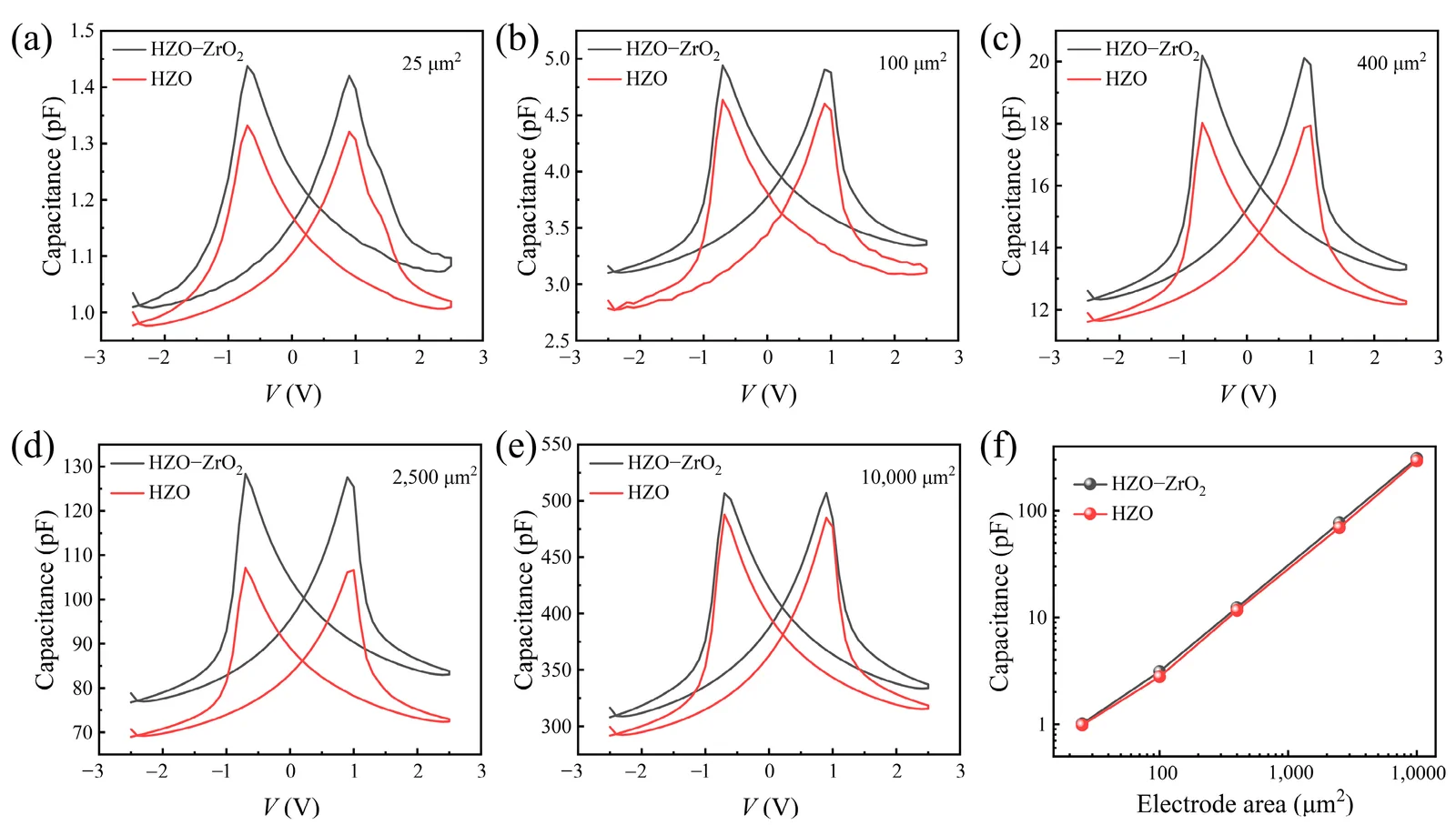
Area-dependent capacitance–voltage characteristics measured at 100 kHz. (**a**–**e**) Curves for 25, 100, 400, 2500, and 10,000 μm^2^ devices, respectively. (**f**) Capacitance at 2.5 V, replotted from the numerical values in Table 1. Connecting lines guide the eye and are not fitted scaling laws.

The capacitance–voltage curves in Figure 4 exhibit the characteristic two-peak, butterfly-like response in both stacks. Such behavior is consistent with enhanced differential response near polarization reversal. At the common positive-bias comparison point, *C*_2.5_ increases strongly with electrode area. The HZO values are 1.0, 3.0, 12.0, 72.0, and 310.0 pF, whereas the HZO–ZrO_2_ values are 1.1, 3.3, 13.8, 82.0, and 345.0 pF. The corresponding increases are 10.0%, 10.0%, 15.0%, 13.9%, and 11.3%, respectively. Table 1 provides the numerical basis for the subsequent analysis.

*C*_2.5_ is the measured 100 kHz capacitance at 2.5 V. Reductions are calculated relative to HZO. Single-record values with a numerical resolution of 0.1 ns. The estimated single-reading timing uncertainty is *σ*_t_ ≈ 0.1 ns (50–100 ps sampling interval; ≈0.7 mV RMS baseline noise at 50 mV/div); the 0.1 ns differences at 25 and 100 μm^2^ are within this uncertainty.

For the three larger areas, the capacitance per area is approximately 28.8–31.0 fF/μm^2^ for HZO and 32.8–34.5 fF/μm^2^ for HZO–ZrO_2_. At 25 μm^2^, these values rise to 40 and 44 fF/μm^2^. The broad proportionality supports an area-dependent capacitive contribution, while the smallest-device deviation makes fringe and pad contributions relevant possibilities. It would be premature to assign the entire deviation to a change in material permittivity. The available five-point series supports approximate scaling, not a precision determination of parasitic capacitance or a universal area law.

A higher total capacitance after adding a layer cannot be explained by inserting an otherwise passive series dielectric while leaving every original component unchanged. In that restricted picture, reciprocal capacitances add and the stack capacitance decreases. The measured increase therefore suggests that insertion also modifies the effective dielectric response, phase mixture, or interfacial boundary conditions, or that measurement-dependent contributions differ. Seed-layer studies make structural modification plausible [10,11,12]. However, the capacitance result by itself does not distinguish those possibilities or prove an increase in the intrinsic dielectric constant of the HZO component.

The bias at which capacitance is evaluated is also consequential. The selected 2.5 V point is away from the principal peaks and provides a consistent operational comparison, but the capacitor passes through other biases before switching begins. Its differential capacitance along that trajectory need not equal *C*_2.5_. Moreover, a 100 kHz small-signal experiment probes a different timescale and perturbation amplitude from a nanosecond pulse. Substituting its capacitance into a transient model is therefore an approximation to be tested. Agreement of area trends is informative, but does not by itself validate that substitution quantitatively.

### 3.3. Switching-Onset Delay Decreases in the Interlayer Capacitors

**Figure 5:**
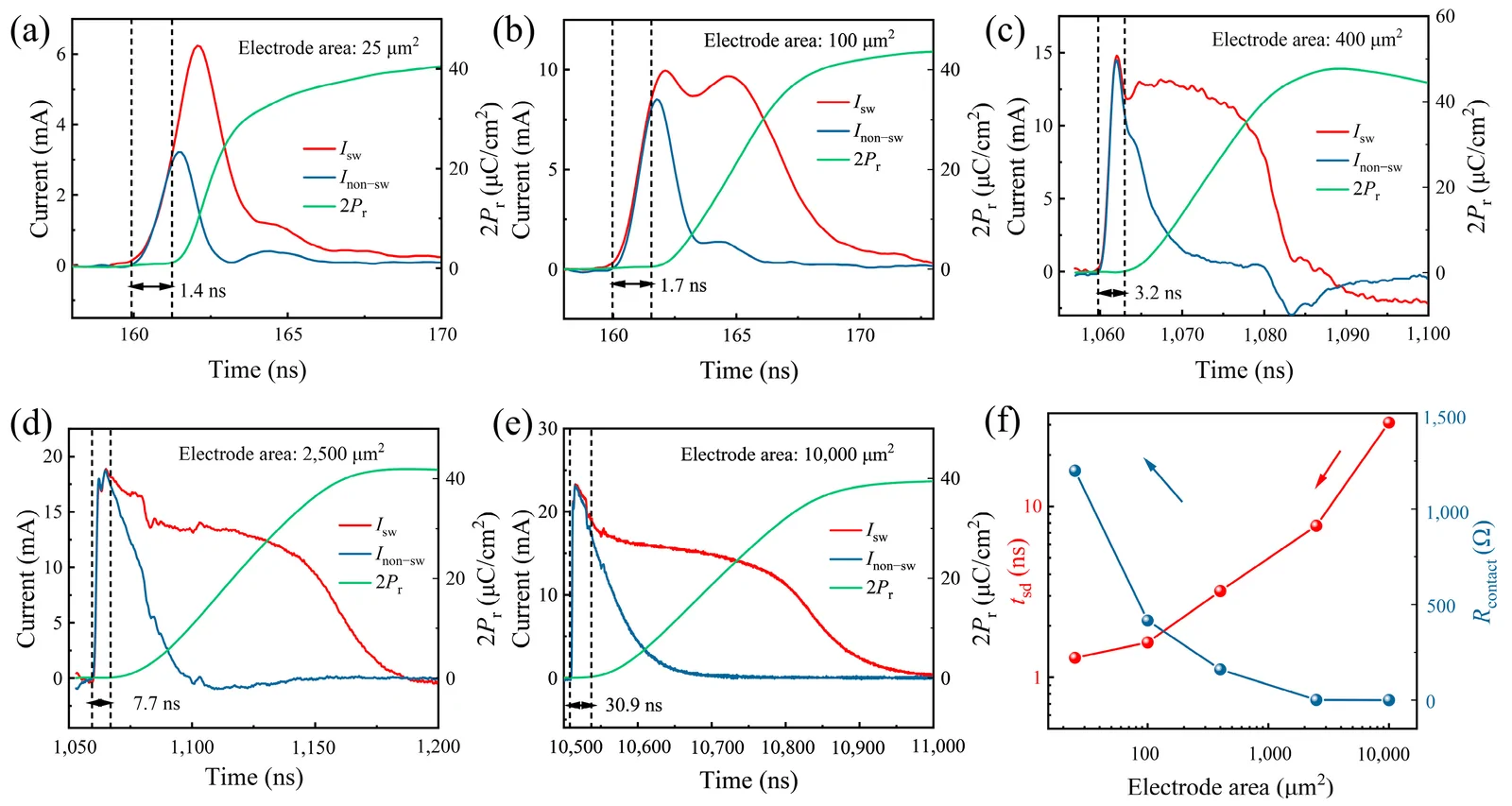
Transient responses of HZO capacitors. (**a**–**e**) Switching current *I*_sw_, non-switching current *I*_non-sw_, and the polarization trace labeled 2*P*_r_ for areas of 25, 100, 400, 2500, and 10,000 μm^2^, respectively. Dashed markers indicate the operational delay interval *t*_sd_. (**f**) Area-dependent onset delay replotted from Table 1. The marked delay excludes the subsequent polarization rise; no intrinsic switching time is extracted.

The HZO current transients in Figure 5 show an initial response shared approximately by switching and non-switching events, followed by polarization-associated divergence. Their marked delays increase with area from 1.4 to 30.9 ns. The increase is particularly strong between 2500 and 10,000 μm^2^, where *t*_sd_ changes from 7.7 to 30.9 ns. This behavior is consistent with the larger charge required to raise the voltage across a larger capacitor. The time-axis offsets in the individual records do not enter the comparison; *t*_sd_ is an interval within each record.

**Figure 6:**
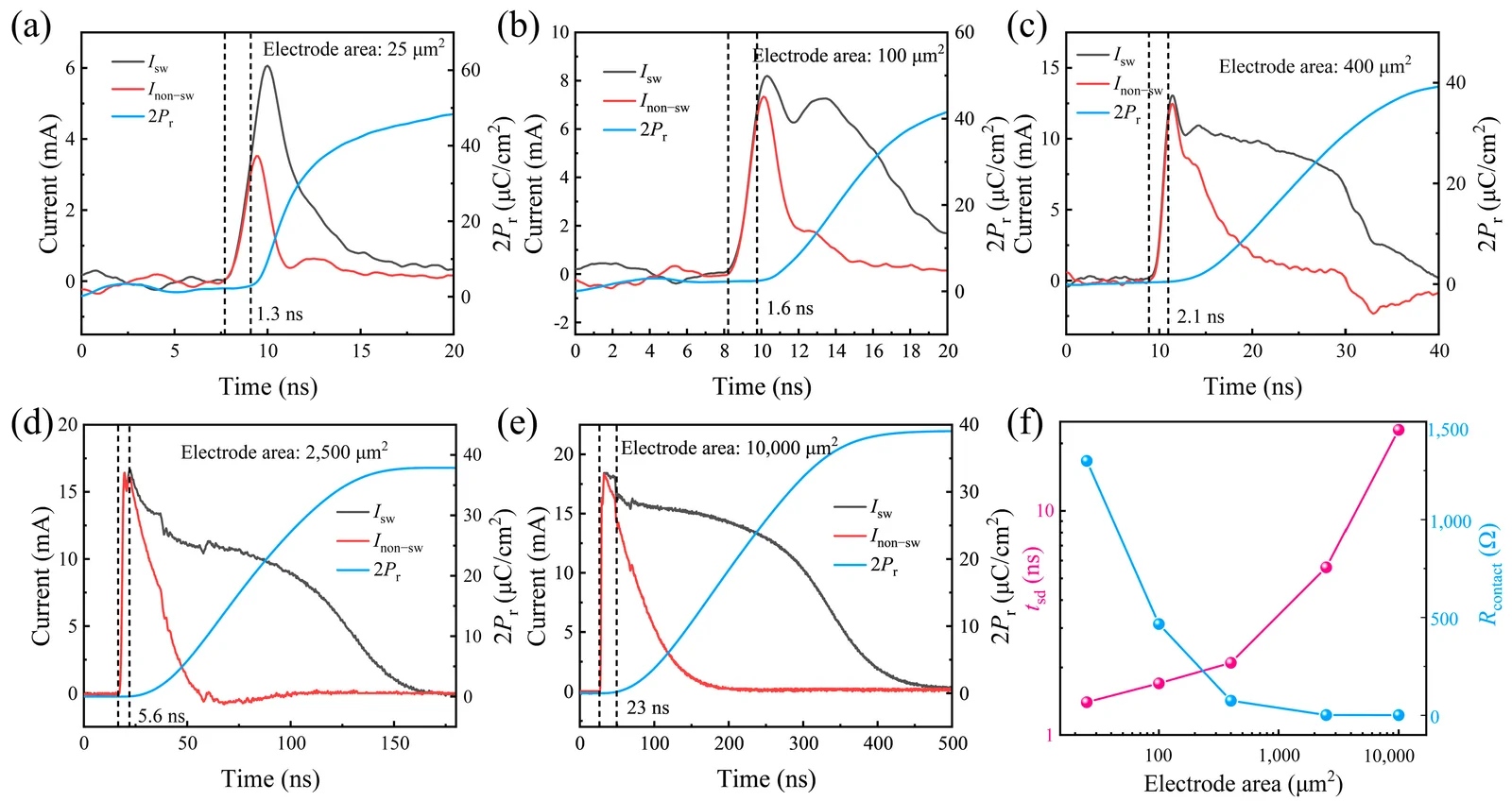
Transient responses of HZO–ZrO_2_ capacitors. (**a**–**e**) *I*_sw_, *I*_non-sw_, and the polarization trace labeled 2*P*_r_ for 25, 100, 400, 2500, and 10,000 μm^2^ devices, respectively. Dashed markers delimit the operational onset delay. (**f**) Delay replotted from Table 1. Line colors in the experimental panels follow their respective legends.

The HZO–ZrO_2_ transients in Figure 6 show the same qualitative ordering with area, but their marked delays are lower at every matched area. The values are 1.3, 1.6, 2.1, 5.6, and 23.0 ns. Relative to HZO, the absolute reductions are 0.1, 0.1, 1.1, 2.1, and 7.9 ns. The corresponding relative reductions are 7.1%, 5.9%, 34.4%, 27.3%, and 25.6%. Thus, the largest absolute benefit occurs at 10,000 μm^2^, whereas the largest relative benefit occurs at 400 μm^2^. The comparison should not be compressed into a single area-independent improvement factor.

The lower delays are observed despite the higher measured capacitances. Therefore, capacitance reduction cannot account for the interlayer trend. Some combination of the effective charging impedance, switching threshold, dynamic capacitance, and polarization-onset behavior must differ. At the two smallest areas, the 0.1 ns separation is modest and requires repeated, calibrated measurements before statistical significance can be assigned. Moreover, the polarization continues to evolve after the marked onset in both stacks. At the two smallest areas, the 0.1 ns separation is within the estimated single-reading timing uncertainty (*σ*_t_ ≈ 0.1 ns) and of the order of the sampling interval (50–100 ps); it therefore cannot be assigned statistical significance and is reported for completeness only. The quantitative delay-reduction claim of this work rests on the three larger areas, where the absolute reductions of 1.1, 2.1, and 7.9 ns exceed *σ*_t_ by factors of 11–79.

### 3.4. Effective Resistance Metrics and Failure of the Unity-Factor Inversion

**Figure 7:**
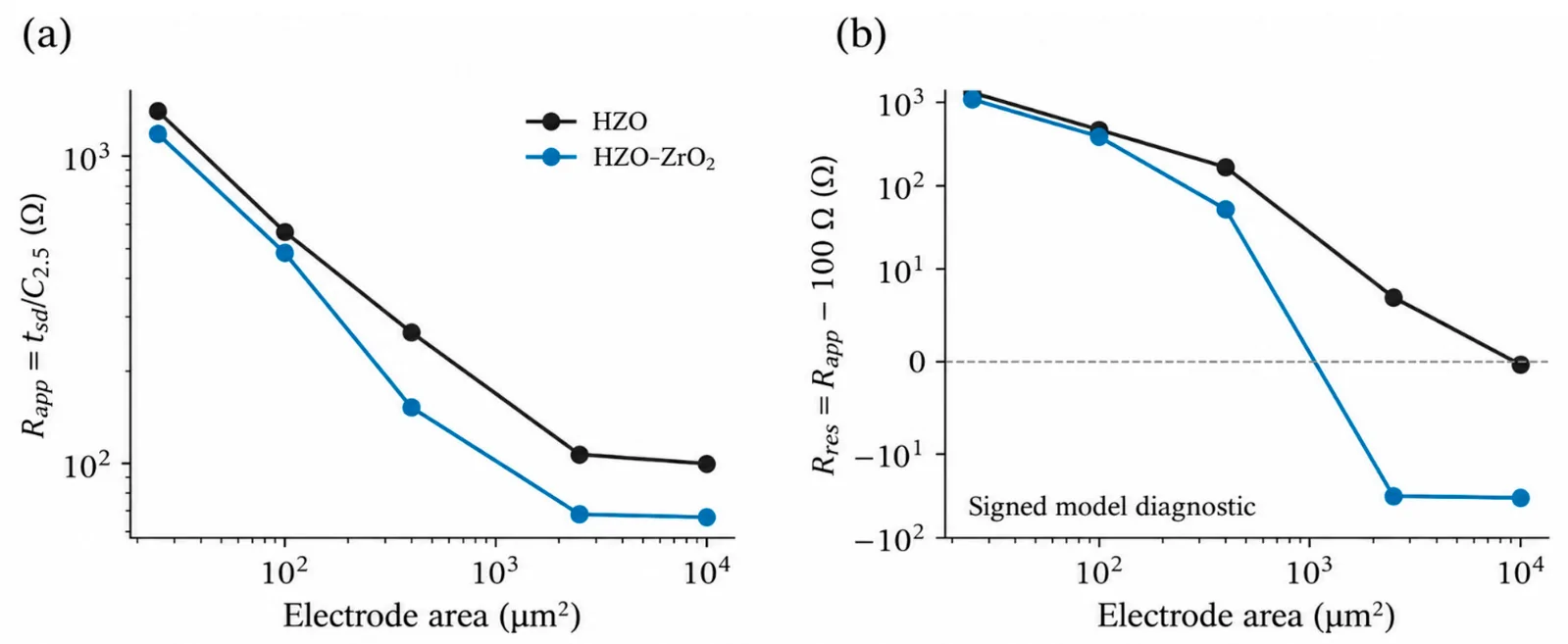
Comparison of effective transient metrics calculated from Table 1. (**a**) *R*_app_ = *t*_sd_/*C*_2.5_. (**b**) Signed unity-factor residual *R*_res_ = *R*_app_ − 100 Ω; a symmetric logarithmic vertical scale retains negative values, with a linear region within ±10 Ω. The dashed line indicates zero. Neither metric is an independently measured contact resistance. Connecting lines guide the eye.

Figure 7 compares the derived effective metric *R*_app_ and its unity-factor residual. For HZO, *R*_app_ is 1400.0, 566.7, 266.7, 106.9, and 99.7 Ω in ascending area order. For HZO–ZrO_2_, the corresponding values are 1181.8, 484.8, 152.2, 68.3, and 66.7 Ω. These values consistently decrease with increasing area and are lower for the interlayer stack. This is a useful comparative result, provided *R*_app_ retains its operational definition and is not relabeled as an independently measured contact resistance.

Subtracting the nominal 100 Ω external contribution gives HZO residuals of 1300.0, 466.7, 166.7, 6.9, and −0.3 Ω. The HZO–ZrO_2_ residuals are 1081.8, 384.8, 52.2, −31.7, and −33.3 Ω. The near-zero HZO value at the largest area is sensitive to rounding. In contrast, the negative interlayer residuals are too large to be reconciled by the displayed numerical rounding alone. Table 2 reports them explicitly as model-consistency diagnostics. The same arithmetic cannot support positive, small contact-resistance values at those points without changing a stated input or assumption.

The threshold factor provides a simple physical explanation for why a delay can be shorter than 100 Ω × *C*_2.5_ without any negative circuit element. Under the idealized assumptions *C* = *C*_2.5_ and *R*_tot_ ≥ 100 Ω, the 10,000 μm^2^ interlayer result requires *α* ≤ 0.667. This corresponds to *V*th/*V*_step_ ≤ 0.487. The calculation is a conditional bound, not a measured threshold. It shows why setting *α* to unity is an additional assumption rather than a general identity, and why a faster onset can reflect a changed switching threshold as well as a changed series impedance.

For illustration, an ideal capacitor reaches half of its final step voltage after 0.693 *RC*, whereas reaching approximately 63.2% requires one *RC*. The two definitions differ even for the same resistor and capacitor. A ferroelectric onset criterion adds material dependence because its threshold can vary with polarization history and pulse conditions. Consequently, agreement between delay and *RC* to within the same order of magnitude is insufficient evidence for extracting a small difference between two large resistances. The numerical inconsistency exposed here is therefore a limitation of the inversion, not a contradiction of the measured faster onset.

## 4. Discussion

### 4.1. Interfacial Contributions to the Observed Delay Reduction

The combined observations establish an association between ZrO_2_ insertion and faster switching onset within the investigated measurement configuration. A structural route is credible because ZrO_2_ seeding can influence crystallization and the population of electrically active orientations [10,12]. The sharper loop transition is compatible with such a change. An electrical route is also credible because interface chemistry can modify screening and the voltage distribution [7]. However, these routes are coupled, and neither is uniquely selected by the present capacitance and delay measurements.

It is especially important to distinguish the buried tungsten/oxide interface from the external probe/tungsten contact. The ZrO_2_ layer modifies the former by design; its insertion does not directly demonstrate improved contact between a probe tip and a metal pad. A series-circuit fit can collect multiple contributions into one effective parameter, but cannot locate them spatially. Claims of reduced contact resistivity would require a separately validated electrical extraction and an appropriate geometry. The present result is more securely described as reduced operational delay associated with a modified capacitor stack.

Related literature also shows that interface optimization is not equivalent to a universal reliability improvement. HZO–ZrO_2_ superlattices have been designed to suppress charge injection and enhance endurance [28], while oxygen-active CeO_2_-based interfaces provide another approach to fatigue control [29]. These studies involve distinct structures and direct cycling evidence. Their endurance or energy results cannot be transferred to the approximately 1 nm ZrO_2_ insertion examined here. Recent equivalent-interface analyses further emphasize the relevance of interfacial contributions to HZO switching [30], but do not remove the need to validate the parameters of each measurement configuration.

### 4.2. What Lateral Scaling Can and Cannot Establish

A commonly proposed scaling picture assumes a capacitance *C* = *cA* and an additional resistance *R*_add_ = *r*/*A*, where *c* is capacitance per area and *r* is an area-normalized resistance parameter. If *α* remains constant, Equation (2) then gives *t*_sd_ = *α*(*R*_ext_*cA* + *rc*). The first term decreases with area, whereas the second approaches an area-independent contribution. This conditional model explains why reducing area may yield diminishing delay improvements. It does not establish that the asymptote originates from a contact or that the inverse-area resistance law holds in the present devices.

The measured delays change only modestly between 100 and 25 μm^2^, consistent with diminishing returns over that interval. Yet similar behavior could arise from the source rise time, an onset-detection floor, distributed wiring, or polarization nucleation. Since the smallest structures are still 5 μm in equivalent side length, the data do not establish a scaling limit for nanoscale memory cells. Published nanoscale electrode studies [14] and optimized transient structures [18] operate in different geometrical and electrical regimes. Extrapolation must account for those differences rather than assume that the observed microscale trend persists indefinitely.

A lower onset delay does not by itself determine memory write latency, but its role in ultrashort-pulse operation deserves emphasis. In a high-speed write operation, a pulse of fixed width tpulse is applied, and the write succeeds only if *t*_pulse_ ≥ *t*_sd_ + *t*_sw_, where *t*_sw_ is the additional time needed to reach the switched-polarization fraction required by the sense margin. The charging interval *t*_sd_ is an unavoidable part of this pulse budget: no switched charge is delivered until the ferroelectric voltage reaches the coercive voltage. Reducing *t*_sd_ therefore enlarges the fraction of the pulse available for reversal. As a numerical illustration based on Table 1, for a 5 ns write pulse applied to the 400 μm^2^ devices, the time remaining for reversal grows from 1.8 ns (HZO) to 2.9 ns (HZO–ZrO_2_), an increase of about 60%; at 10,000 μm^2^, the corresponding gain is 7.9 ns. In the short-pulse limit, the effect is binary: if *t*_sd_ exceeds the pulse width, no reversal occurs at all, irrespective of the intrinsic switching kinetics. The reversal time *t*_sw_ itself depends on the electrode area (the number and size of switching domains), the coercive-voltage distribution, the applied field, and the defect landscape [16,25,26,27]. Resolving it requires pulse-amplitude- and pulse-duration-dependent measurements [16,17] and lies beyond the present scope. The practical implication is to treat *t*_sd_ and *t*_sw_ as separately optimizable quantities: the onset delay reported here characterizes the electrical-delivery component of the write time, which is the component directly controlled by stack and interface engineering. A functional write operation must deliver sufficient switched charge, satisfy the required state-separation margin, and preserve reliability over cycling. The transient tails in Figure 4 and Figure 5 make clear that onset and completion are distinct observables. For circuit design, the practical implication is to co-optimize stack response and drive delivery, then evaluate a defined switched-polarization target. It is not sufficient to minimize one fitted time constant while leaving the state reached by the pulse unspecified.

### 4.3. Requirements for a Quantitative Resistance Extraction

A calibrated extraction would first establish the source waveform and device voltage at the relevant reference plane, including the distinction between programmed voltage, matched-load voltage, and open-circuit source amplitude. A non-switching reference over the relevant frequency range could then constrain the dynamic charging response. Repeating the measurement with several known external series resistances would test whether the delay follows the expected dependence while separating a fixed instrumental contribution. These measurements would make the contact-resistance interpretation testable rather than dependent on the assumption *t*_sd_ = *RC*.

The same analysis should propagate uncertainty. In the unity-factor diagnostic, *R*_app_ depends directly on delay and inversely on capacitance. For uncorrelated small uncertainties, its variance is approximately (*δt*/*C*)^2^ + (*tδC*/*C*^2^)^2^. Subtracting 100 Ω can make the fractional uncertainty of a small residual very large, even when the parent measurements are reasonably precise. In the threshold model, uncertainty in *α* introduces another contribution. Accordingly, the signs and magnitudes in Table 2 are useful for detecting inconsistency, but small residual differences should not be assigned high precision or interpreted as specific contact resistivities.

A further check concerns the difference between the first detectable switching event and a chosen fractional polarization level. Noise can move a graphical onset, especially when the initial polarization increment is small. Applying a reproducible threshold to repeated traces, reporting the timing reference, and comparing both polarities would determine whether the small-area separation is stable. Measurements on multiple devices would also distinguish variation within a stack from differences between stacks. These requirements are particularly relevant to the 0.1 ns differences, whereas the larger-area records show a more pronounced numerical separation under the stated analysis.

No activation field, nucleation-time distribution, or intrinsic reversal time is fitted here. Those quantities require waveform information over suitable pulse amplitudes and durations, as illustrated by prior kinetic studies [16,17]. Likewise, the reconstructed charge should be checked against an independent remanent-polarization measurement before using a short-pulse trace to specify a memory state. This restrained interpretation preserves the primary comparison while identifying the additional observables needed to turn an effective stack-level improvement into a mechanistically resolved design rule.

The general principle of separating device dynamics from electrical delivery is relevant to other memory technologies. Nevertheless, the polarization-threshold model used here is not a validated extraction method for resistive, magnetic, or phase-change memories. Those devices involve different state variables and switching pathways. Extension would require an appropriate equivalent circuit, a measured dynamic impedance, and an independently tested relationship between stimulus and switching onset. The transferable contribution is the consistency-checking framework, not a universal numerical formula for all memory contacts.

## 5. Conclusions

Approximately 10 nm HZO capacitors containing an approximately 1 nm bottom ZrO_2_ interlayer exhibit lower measured switching-onset delays than the reference capacitors at areas of 400–10,000 μm^2^, with absolute reductions of 1.1–7.9 ns; at 25 and 100 μm^2^, the differences (0.1 ns) are within the estimated timing uncertainty of ≈0.1 ns. The electrical results and local structural images support interlayer-associated modification of the operational response. However, inversion using *t*_sd_/C − 100 Ω becomes inconsistent for the larger interlayer devices. Because the onset delay measures the *RC* charging time to the switching threshold, its reduction enlarges the fraction of an ultrashort write pulse that remains available for polarization reversal and can determine whether a short write pulse succeeds at all. A threshold-dependent charging factor and measurement calibration are therefore necessary before assigning a physical contact resistance. These findings identify a useful direction for stack and drive optimization while distinguishing an experimentally observed delay benefit from an unverified microscopic resistance mechanism.

## Figures and Tables

**Table 1:** Measured capacitance and switching-onset delay with relative delay reduction.

Area (μm^2^)	*C*_2.5_ HZO (pF)	*C*_2.5_ HZO–ZrO_2_ (pF)	*t*_sd_ HZO (ns)	*t*_sd_ HZO–ZrO_2_ (ns)	Delay Reduction (%)
25	1.0	1.1	1.4	1.3	7.1
100	3.0	3.3	1.7	1.6	5.9
400	12.0	13.8	3.2	2.1	34.4
2500	72.0	82.0	7.7	5.6	27.3
10,000	310.0	345.0	30.9	23.0	25.6

**Table 2:** Recomputed effective metrics and signed residuals under the unity-factor assumption.

Area (μm^2^)	*R*_app_ HZO (Ω)	*R*_app_ HZO–ZrO_2_ (Ω)	*R*_res_ HZO (Ω)	*R*_res_ HZO–ZrO_2_ (Ω)
25	1400.0	1181.8	1300.0	1081.8
100	566.7	484.8	466.7	384.8
400	266.7	152.2	166.7	52.2
2500	106.9	68.3	6.9	−31.7
10,000	99.7	66.7	−0.3	−33.3

*R*_res_ is a diagnostic residual, not a physical contact resistance. Negative values identify inconsistency of the unity-factor *RC* inversion.

## Data Availability

The raw data supporting the conclusions of this article will be made available by the authors on request.
